# Tuning the MPI‐ESM1.2 Global Climate Model to Improve the Match With Instrumental Record Warming by Lowering Its Climate Sensitivity

**DOI:** 10.1029/2019MS002037

**Published:** 2020-05-01

**Authors:** Thorsten Mauritsen, Erich Roeckner

**Affiliations:** ^1^ Department of Meteorology Stockholm University Stockholm Sweden; ^2^ Max Planck Institute for Meteorology Hamburg Germany

**Keywords:** Climate, Modeling, Tuning

## Abstract

A climate model's ability to reproduce observed historical warming is sometimes viewed as a measure of quality. Yet, for practical reasons it cannot be considered a purely empirical result of the modeling efforts because the desired result is known in advance and so is a potential target of tuning. Here we report how the latest edition of the Max Planck Institute for Meteorology Earth System Models (MPI‐ESM1.2) atmospheric component (ECHAM6.3) had its sensitivity systematically tuned in order to improve the modeled match with the instrumental record. In practice, this was done by targeting an equilibrium climate sensitivity of about 3 K, slightly lower than in the previous model generation (MPI‐ESM), which warmed more than observed, and in particular by addressing a climate sensitivity of about 7 K in an intermediate version of the model. In the process we identified several controls on cloud feedback, some of which confirm recently proposed hypotheses. We find the model exhibits excellent fidelity with the observed centennial global warming. We further find that an alternative approach with high climate sensitivity compensated by strong aerosol cooling instead would yield colder than observed results in the second half of the twentieth century.

## Key Points

- We document how the MPI‐ESM1.2 was tuned to the instrumental record warming
- The tuning involved lowering the models climate sensitivity

## 1 Introduction

Global climate models are tools that see broad application in the climate sciences and beyond, however, poorly documented decisions made during their development often complicate the interpretation of the results and limit the knowledge that can be gained from climate model experiments. Contemporary climate models are tuned, foremost with the purpose of stabilizing their global mean temperature at a reasonable level (Hourdin et al., 2017; Mauritsen et al., 2012). Broadly speaking, tuning can be thought of as changes made to the model in order to obtain certain properties, and without tuning climate models would drift away from the observed state of the Earth's climate. Typically, tuning consists of adjusting a set of model parameters toward the end of a development cycle, but could be generalized to be any changes made to the model that are in some way guided by the model results.

The ability to represent the warming over the industrial era is sometimes considered a key benchmark of climate model quality. Indeed, a somewhat naive assertion is that a model used to make future projections should be able to match past warming. However, the instrumental record is driven by a multitude of forcing agents, foremost warming by greenhouse gases and a highly uncertain compensating cooling by anthropogenic aerosol particles. The latter has stabilized to some extent since the 1970s and is unlikely to continue to increase into the future as air quality regulations aim at reducing aerosol emissions, primarily for health reasons. Thus, whereas a low climate sensitivity in a model can be paired with a weak aerosol cooling, or vice versa, in order to obtain an overall warming in agreement with the instrumental record (Kiehl, 2007), this type of compensation is less evident in future projections wherein long‐lived greenhouse gases, foremost CO_2_, dominates. As such, a reasonable match with the instrumental record may be regarded as a necessary, but insufficient test for climate model projections into the future.

It is not likely that modeling centers in general have applied explicit tuning practices to improve their historical simulations in the past: Even if some early results (Kiehl, 2007) would suggest otherwise, more recent simulations exhibit less signs of deliberate compensation (Forster et al., 2013). In a survey among 23 modeling centers, 35% replied that twentieth century warming was an important target for their model development, while 30% would not consider it at all during their development (Hourdin et al., 2017); the latter view was expressed in a smaller independent survey among six centers by Schmidt et al. (2017), whereas Zhao et al. (2018) explained how at the Geophysical Fluid Dynamics Laboratory the tuning of both aerosol forcing and sensitivity was done considering the instrumental record. Regardless of point of view, though, it is imperative that modeling centers document to which extent their decisions were influenced by the instrumental record.

When we were faced with a model system that was bound to fail at reproducing the instrumental record warming, we chose an explicit approach were the past temperature trend is a tuning target. The aim of this paper is to explain how we conducted the tuning in the latest version of the Max Planck Institute for Meteorology climate models in order to improve the match to the instrumental record warming (sections 2 and 3), and subsequently, we explain what we learned about cloud feedbacks during these tuning efforts (section 4). We then investigate how well we managed against observations of global mean surface temperature (section 5) and provide some concluding remarks (section 6).

## 2 Background

The previous generation of the Max Planck Institute Earth System Model (MPI‐ESM, Stevens et al., 2013; Giorgetta et al., 2013) was applied among many other things to conduct simulations during the fifth phase of the Coupled Model Intercomparison Project (CMIP5). After the fact, we identified a number of programming errors impacting the conservation of energy and the representation of partial cloud fractions in the atmospheric physics parameterizations (convection, clouds, and turbulence) of the atmospheric component; the nature and remedy of which is described in Mauritsen et al. (2019). The resulting corrected ECHAM6.2 model was finalized as a stand‐alone atmosphere model in late October of 2013. Shortly thereafter, we found that relative to the predecessor the new model had an approximately doubled equilibrium climate sensitivity (ECS) of about 7 K. This was a result of rapidly dissipating tropical low‐level clouds with warming in the updated model; these clouds mostly reflect sunlight back to space and so the result is a positive cloud feedback. We then found that for instance increasing the lateral entrainment rate for shallow convection could largely eliminate the increase in climate sensitivity. The finding was informed by studies that were not yet published at the time concerning low‐level convective mixing processes and cloud feedbacks (Brient et al., 2016; Sherwood et al., 2014; Zhao, 2014).

We were now faced with a dilemma. Whereas previous studies had shown that the ECHAM6.1 models climate sensitivity was fairly insensitive to changing typical tuning parameters (Mauritsen et al., 2012), the found parametric dependency could no longer simply be ignored. Furthermore, we knew that the MPI‐ESM already warmed more than what is observed during the twentieth century (Giorgetta et al., 2013), and doubling the equilibrium climate sensitivity would certainly act to make this issue worse. At the time we had neither plans to include additional aerosol cooling effects, which could have been tuned to offset some of the warming (Golaz et al., 2013), a tunable aerosol forcing only later became available (Fiedler et al., 2017; Stevens et al., 2017), nor was it an option to roll back any of the corrections made to the physics in ECHAM6.2, which were clearly desirable.

We therefore decided that the goal of further tuning was to improve the models representation of the twentieth century warming and in practice this was done by explicitly tuning down the climate sensitivity. As a tuning target we somewhat conservatively decided to aim at and ECS of 3 K, slightly below the 3.5 K of the predecessor MPI‐ESM. During a period, as shall be described below, we were faced with difficulties in matching this target and therefore discussed not accepting a value higher than 4 K. As we did not manage to create a model version with much less than 3 K sensitivity, it was not necessary to consider a lower acceptance limit, but supposedly, we would not have accepted a model below 2 K and would have preferred to stay above 2.5 K, as at the time we would have probably deemed it would warm too little. It is noteworthy that the target ECS expresses our collective experience as of the year 2014 when the retuning was conducted and as such was neither based on deep or elaborate considerations of the quantitative connection to historical warming nor the true value of Earth's climate sensitivity. We shall return to these aspects in more detail in sections 5 and 6.

## 3 A Practical Procedure to Tune Climate Change Feedback

**Figure 1:**
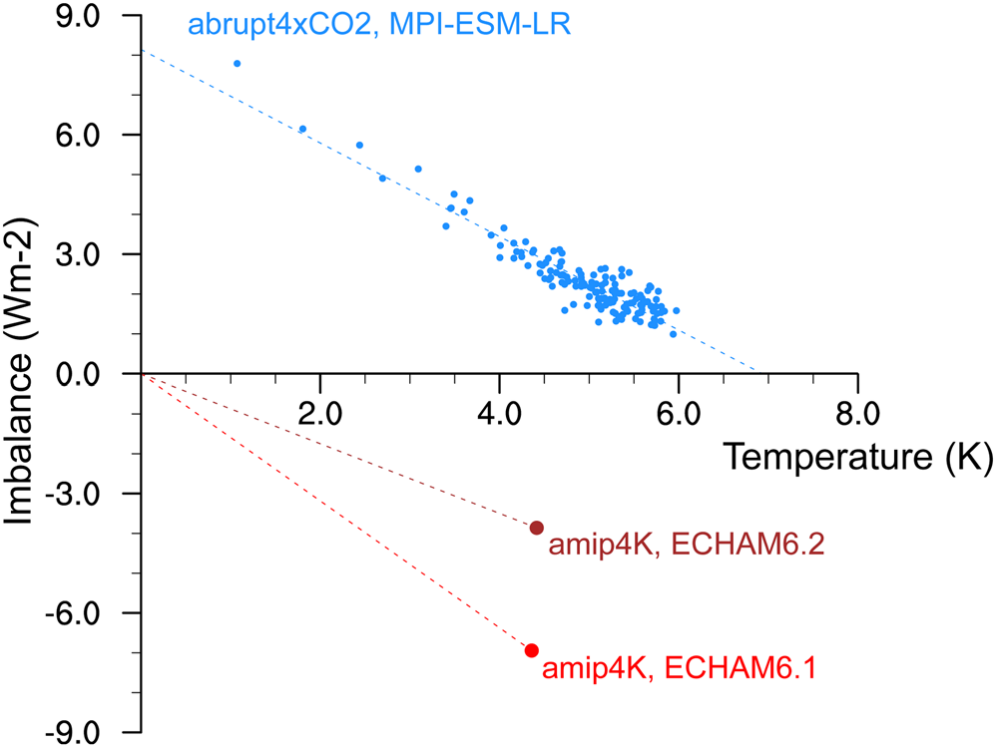
Estimation of climate sensitivity by means of equation (1). Individual years of the coupled model experiment with abruptly quadrupled CO_2_ is shown as blue markers and a linear regression as a dashed line. Red and brown markers are the 30‐year averages in *amip4K* experiments with uniformly raised SSTs in two versions of ECHAM.

Equilibrium climate sensitivity of a coupled climate model is today de facto measured using an idealized forcing run of 150 years wherein CO_2_ is abruptly quadrupled starting from a stationary state control simulation (*abrupt4xCO2*, Figure 1,  Andrews et al., 2012). These runs are not long enough for the deep oceans to equilibrate with the radiative forcing, and so a linear regression of top‐of‐atmosphere imbalance against global mean surface temperature change is typically used to estimate the magnitude of the equilibrium warming (Gregory et al., 2004). The resulting intercept is divided by two to get the ECS for a single CO_2_ doubling: in case of MPI‐ESM‐LR then ECS ≈ 3.5 K. The procedure is, however, prohibitively slow because it involves a spin‐up of the coupled ocean‐atmosphere model which takes hundreds to thousands of simulation years, or in practice weeks to months of real time. Thus, under the time constraints of typical model development cycles tuning ECS systematically using the *abrupt4xCO2* experiment would hardly be feasible.

To overcome this limitation, we took advantage of the method suggested by Cess et al. (1989) wherein an atmosphere‐only model is run with uniformly raised SSTs as surface boundary conditions (*amip4K*). The resulting change in top‐of‐atmosphere radiation imbalance (*N*), relative to that in a reference simulation (*amip*), can be interpreted as climate change feedback, *λ*≈Δ*N*/Δ*T*. However, for a number of reasons this estimate of *λ* does not necessarily equal that obtained in the *abrupt4xCO2* experiment, for instance, the setup lacks polar amplification and the sea ice is kept fixed. To account for this inaccuracy, we used the known feedback in such experiments (*λ*_6.1_) and the climate sensitivity (ECS_6.1_) from ECHAM6.1/MPI‐ESM‐LR to estimate that in subsequent model versions:

(1) $$\text{ECS}\approx {\text{ECS}}_{6.1}\cdot \frac{\lambda_{6.1}}{\lambda },$$

whereby it is assumed that relative changes in *λ* in the Cess experiment carry over to those in the coupled model experiment and that the radiative forcing of CO_2_ does not change. Figure 1 shows that *λ* in ECHAM6.2 is about half as large as that of ECHAM6.1, and so our estimate is that ECS of the former is about twice that of the latter, close to 7 K.

We found that running the model for 10 years was more than sufficient for our purposes to average out internal weather‐induced variability in the radiation balance which could impact the estimate of *λ*. Because the ECHAM model integrates quite fast with limited resources, the two experiments required to estimate ECS (*amip*, *amip4K*) can be done in parallel and, conveniently, over night. Presumably, more accurate ECS estimates can be obtained by using patterns of warming and sea ice melt from a previous coupled simulation (Gettelman et al., 2012), assuming these will not change in response to the cloud feedback changes, or by applying a mixed‐layer ocean as a surrogate for a fully coupled ocean model. The latter would take considerably longer and slow down progress, with no obvious gain.

**Figure 2:**
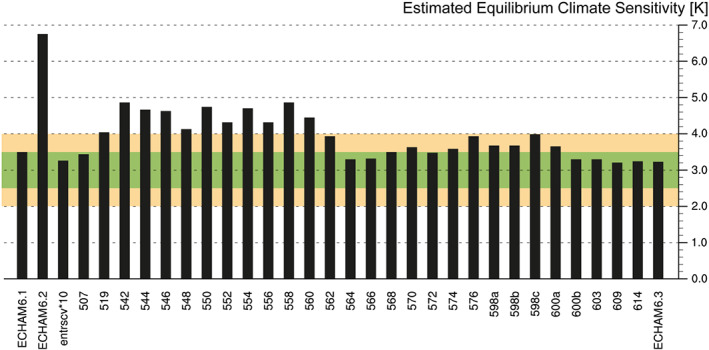
Evolution of estimated ECS using equation (1) as it was monitored during the development from ECHAM6.1 to ECHAM6.3. Three‐digit numbers are shortened MPI‐internal experiment identifiers, for example, mbe0507. Green and yellow bands show our target acceptance ranges which are centered at 3 K as discussed in section 2. Notes taken during experimentation documenting the settings and feedback in each experiment is provided in supporting information Tables S1–S4.

The simple methodology allowed us to systematically monitor and tune the climate sensitivity during the development of ECHAM6.3 (Figure 2). After the initial tests with tenfolded lateral entrainment rate for shallow convection (experiment entrscv*10), as well as various other changes, it was decided to tune and spin‐up the coupled model. During this development stage the estimated ECS had risen again to nearly 5 K (Experiment 542); an estimate that was confirmed by running an *abrupt4xCO2* experiment with the coupled model yielding an ECS of 4.8 K. A period followed wherein we identified which parameters were responsible for the rise in ECS (Experiments 544–562), and a new coupled model was spun up (experiment 564). The resulting model, however, had issues with too thin sea ice and too little precipitation on tropical lands; issues that we had previously addressed by allowing mixed‐phase clouds to persist longer and by suppressing middle‐ and upper‐level cloud formation, respectively (see section 4 for more explanation). A period seeking a compromise between these three issues ended as we identified an additional control on *λ* by stratocumulus clouds (Experiment 600b), whereby cloud formation is enhanced under an elevated low‐level inversion. With this we were able to obtain a satisfactory solution for the coupled model tuning used in ECHAM6.3, which serves as the atmospheric component of both MPI‐ESM1.1 and MPI‐ESM1.2, and both of these models have the same ECS of about 2.8 K in their LR configurations (Mauritsen et al., 2019).

## 4 Identified Cloud Feedback Controls on Climate Sensitivity

During the tuning of ECHAM6.3 with regard to its climate sensitivity several interesting controls of cloud feedback were identified, some of which we think are worth sharing. The primary controls are related to shallow convection, critical relative humidity in the fractional cloud scheme and mixed‐phase clouds mostly at middle to high latitudes.

The by far most effective control was that of the turbulent lateral entrainment rate for shallow convection. In the applied Tiedtke‐Nordeng moist convection scheme (Nordeng, 1994; Tiedtke, 1989) the turbulent lateral entrainment rate equals the detrainment rate, such that the mass flux stays constant with height, in absence of organized entrainment near the bottom of the parameterized convective cloud and organized detrainmnent near the cloud top. Thus, the entrainment rate parameter controls how strongly the convective updraft is mixed with the environment and vice versa. It is further important to understand that the convective cloud updrafts themselves are not visible to radiation, only the stratiform clouds are.

**Figure 3:**
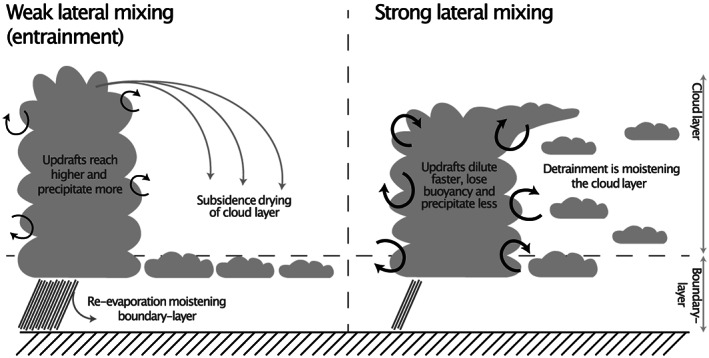
Illustration of the influence of shallow convection lateral entrainment and detrainment on the vertical distribution of clouds. With weak lateral mixing (entrainment, left) as in ECHAM6.2 shallow convective updrafts are less diluted with environmental air and therefore reach higher before losing buoyancy. As a consequence they precipitate more efficiently and act to dry the cloud layer. With stronger lateral mixing as in ECHAM6.3 more humidity is detrained into the cloud layer where as a consequence cloud layers can form. Also, the stronger mixing means the convective updraft loses buoyancy faster and therefore precipitates less efficiently.

**Figure 4:**
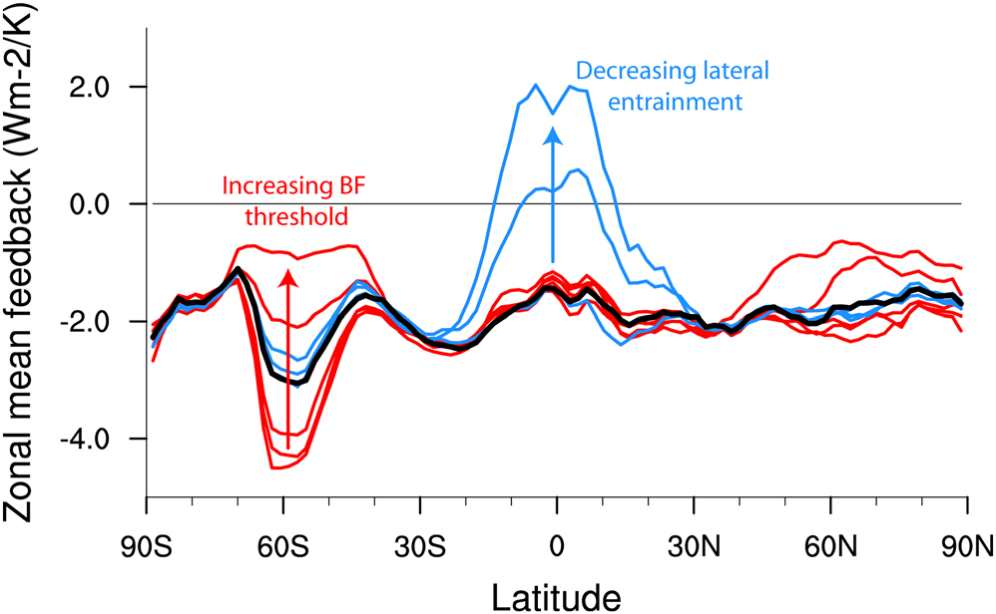
Zonal mean total feedback to globally uniform 4 K SST warming. The black curve shows the result from ECHAM6.3. The lateral entrainment (blue lines) was varied from the default ECHAM6.3 of 3 ·10^−3^ up to 1 ·10^−4^ m^−1^, whereas the cloud ice threshold for the Bergeron‐Findeisen process (red lines) was varied from 1 ·10^−7^ to 5 ·10^−5^ kg m^−3^. In ECHAM6.3 the default threshold is 5 ·10^−6^ kg m^−3^.

In a warmer climate the parameterized shallow convection acts to dry the boundary layer (below about 1 km) and moisten the cloud layer (about 1‐3 km) by enhancing the vertical transport (Sherwood et al., 2014). In ECHAM6.2 the stratiform clouds were almost exclusively situated in the boundary layer (Figure 3, left), and the convection‐induced drying led to a strong reduction of the cloud fraction (see also Nuijens et al., 2015). With the increased lateral entrainment rate, the convection scheme moistens the cloud layer that leads to a more vertically distributed control‐state cloud profile (Figure 3, right). In this case the effect of the convective drying and moistening in a warmer climate is more of a draw between boundary layer decreases and cloud layer increases in cloudiness leading to a smaller trade wind cumulus cloud feedback. Much of the tropics is dominated by marine trade wind cumulus clouds that are parameterized as such shallow convection, and so it is unsurprising that this is where the effect of changing the lateral entrainment rate is largest (Figure 4). Note that the figure show zonal mean total feedback with respect to global warming, which in absence of cloud feedback is about −2 W·m^−2^·K^−1^. Interestingly, the effect seems to saturate for large entrainment rates beyond about 1 ·10^−3^ m^−1^ and so this parameter may have limited effect on models that already have a large entrainment rate.

Perhaps partly related to this, we also found a cloud feedback dependence on the critical relative humidity profile shape (Mauritsen et al., 2019; Sundqvist et al., 1989). The profile determines the level of relative humidity at which sub‐grid scale clouds start forming: the lower the level the more clouds typically form. We found that in particular the critical relative humidity in the free troposphere (parameter *a*_1_ in  Mauritsen et al., 2019) and the parameter controlling the vertical extent of the transition (*a*_3_) from the near‐surface to the free tropospheric value were important for the cloud feedback. Lowering these two parameters led to lower cloud feedback. However, the *a*_1_ parameter had an interesting side effect in that it was almost the only parameter able to control the amount of precipitation on tropical land, which was a major challenge during the development of MPI‐ESM1.2: increasing *a*_1_ led to wetter tropical lands. It is, however, unclear to us how this effect works mechanistically.

More clarity surrounds the effect of the mixed‐phase cloud feedback which can be controlled in ECHAM6.3 using the ice content threshold for activating the Bergeron‐Findeisen effect. In clouds with temperatures between the melting point and around −35 to −40 °C cloud condensate can be either solid or supercooled liquid. However, because the saturation vapor pressure over ice is lower than over liquid at subzero temperatures, ice crystals may grow at the expense of liquid droplets if these are in the vicinity. At the low resolutions applied in models, however, it is necessary to dampen the Bergeron‐Findeisen effect, and in ECHAM this is done using a threshold on the cloud ice content.

In a warmer climate the cloud ice is reduced at the expense of liquid, and because liquid clouds are more reflective than ice clouds, this leads to a negative cloud optical depth feedback. The more ice that exists in the control state, the stronger is this feedback and the lower is the climate sensitivity (Choi et al., 2014). These clouds prevail mostly over the Southern Ocean and at Northern Hemisphere middle to high latitudes where we see the largest impact of changing the parameter (Figure 4). An interesting effect of altering the distribution of liquid and ice in these clouds is that it affects the control‐state sea ice thickness: With a large fraction of ice to liquid in clouds it was difficult to maintain Arctic sea ice volume in the control simulation close to our target. This is because ice clouds are less reflective than liquid clouds and so allows more solar energy absorption in the high latitudes during summer.

It is noteworthy that, whereas lowering the climate sensitivity using the lateral entrainment rate for shallow convection did not mean compromising other aspects of the model, both the critical relative humidity profile and the ice content threshold on the Bergeron‐Findeisen effect were compromises between the need to lower climate sensitivity and other important aspects of the models behavior. Furthermore, since the overall goal of the tuning was to improve historical experiment warming by lowering ECS we applied no constraints on individual feedback mechanisms which may therefore differ from independent estimates.

## 5 Modeled Centennial Warming

**Figure 5:**
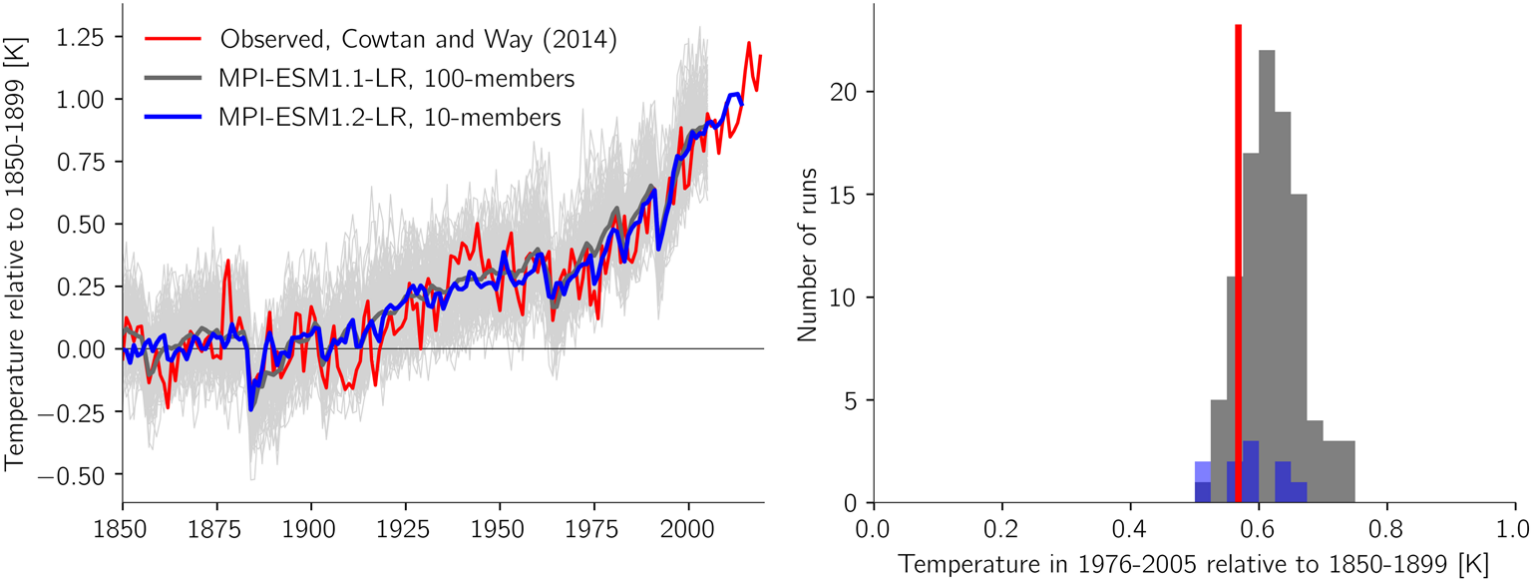
Comparison of the MPI‐ESM1.1‐LR 100‐member ensemble and the MPI‐ESM1.2‐LR 10‐member ensemble *historical* experiments with observed surface temperature. Left is the temporal evolution of global mean temperature with a reference period of 1850–1899. Right panel is showing the distribution of modeled centennial warming defined as 1976–2005 relative to 1850–1899.

The outset for in practice tuning the climate sensitivity through cloud feedbacks in the model, as described above, was a desire to improve the match with instrumental record warming (section 2), and so to verify that we accomplished this goal Figure 5 provides a comparison with observations. Shown is 100 *historical* simulations using the MPI‐ESM1.1‐LR model, also referred to as the grand ensemble (Maher et al., 2019), along with 10 simulations using the MPI‐ESM1.2‐LR model. Both model versions are based on the ECHAM6.3 atmosphere model, share the same ECS, and they mainly differ in terms of their historical forcing which are from CMIP5 and CMIP6, respectively. Here the main difference is that MPI‐ESM1.2‐LR uses the recently developed simple‐plume aerosol parameterization (Fiedler et al., 2017; Stevens et al., 2017).

The runs are compared with the Cowtan and Way (2014) in‐filled HadCRUT data set. The in‐filling procedure of unobserved regions increases the global warming by about 0.1 K compared to the original data set. It is seen that the ensemble means of the two model versions differ fairly little, with slightly less overall warming in MPI‐ESM1.2‐LR, and that on average they track the long term observed global mean temperature very well (right panel). Also, the observed temperature is only occasionally outside the range spanned by the 100 individual ensemble members, as is to be expected if the model exhibits an unbiased mean response and a reasonable amount of internal variability. Thus, the tuned model provides an excellent representation of the observed global warming.

There is, however, many ways in which a model can match the observed centennial warming, foremost by compensating a high climate sensitivity with strong aerosol cooling (Golaz et al., 2013, 2019; Kiehl, 2007). It is possible to estimate the transient warming (*T*) based on bulk model properties as

(2) $$T\approx \frac{-F}{\lambda -\epsilon \gamma },$$

where *F* is the change in total forcing over the period of interest, *ϵ* the ocean heat uptake efficacy (representative of pattern effects), and *γ* is the deep ocean heat uptake coefficient. To arrive at this expression one makes the zero‐layer approximation to the two‐layer Winton‐Held model (Gregory & Forster, 2008; Geoffroy et al., 2013; Held et al., 2010; Jiménez‐de‐la‐Cuesta & Mauritsen, 2019; Winton et al., 2010). From this equation we see that as climate sensitivity increases, meaning the negative feedback parameter *λ* decreases in magnitude, the transient temperature response increases. This may be compensated by larger deep ocean heat uptake, stronger pattern effects, or a weaker forcing. The former two factors are difficult to control, whereas a weaker total forcing can often be achieved through enhanced aerosol indirect effects.

We devise the two‐layer model to investigate how a historical simulation with a 7 K climate sensitivity model might have turned out. We use a version with parameters determined for MPI‐ESM1.2‐LR representing both the pattern effect and state‐dependent feedback,

(3) $$\begin{array}{ccc} C\frac{dT}{dt} & =F+\lambda T+aT^{2}-\epsilon \gamma (T-T_{d}) & \\ C_{d}\frac{dT_{d}}{dt} & =\gamma (T-T_{d}), \end{array}$$

where *F* is a radiative forcing, *T* and *T*_*d*_ the temperatures of the upper and deep layers with respect to an unforced steady state, *C* and *C*_*d*_ the heat capacities of the two layers, *λ*= −1.65 W·m^−2^·K^−1^ is the feedback parameter, *a*=0.04 W·m^−2^·K^−2^ is a quadratic term parameter, *ϵ*=1.2 is the ocean heat uptake efficacy, and *γ*=0.8 W·m^−2^·K^−1^ is the deep ocean heat uptake coefficient. The model parameters were determined from a series of 1,000‐year simulations with 2, 4, 8, and 16 times preindustrial CO_2_ with MPI‐ESM1.2‐LR (Mauritsen et al., 2019). The historical forcing is from Intergovernmental Panel on Climate Change AR5 for the period 1850 to 2011, whereby we have adjusted the forcing for a doubling of CO_2_ from 3.7 to 4.1 W m^−2^, weakened aerosol cooling by 10% in order to peak at −0.65 W m^−2^ relative to 1850 and multiplied the volcanic forcing by 0.7 in order to better match that in the model (Gregory et al., 2016).

**Figure 6:**
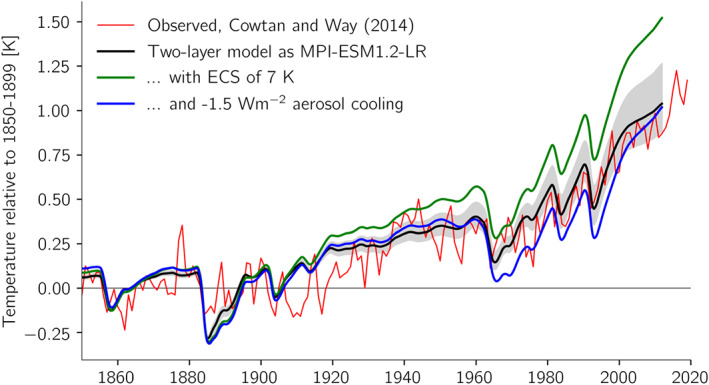
Integrations using the two‐layer model (equation (3)) compared to observed global warming. The base parameters for emulating MPI‐ESM1.2‐LR are taken from Mauritsen et al. (2019), their Table 5. The gray shaded area shows the estimated range of warming for ECS in the range of 2 to 4 K.

**Figure 7:**
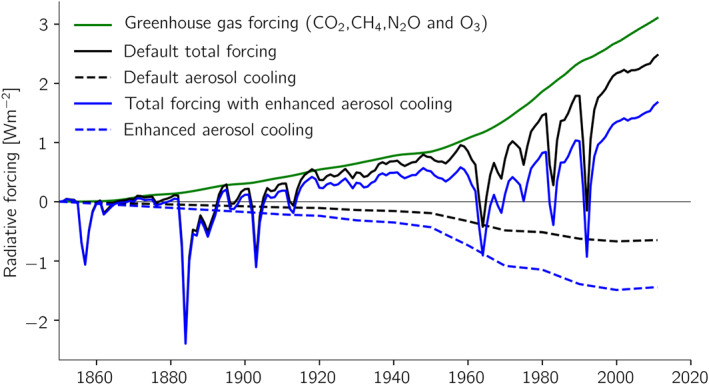
Radiative forcings used in the two‐layer model runs displayed in Figure 6.

We first note how well the two‐layer model matches the behavior of the complex climate model (compare Figures 5 and 6). Also shown in gray is the estimated range for ECS of 2 to 4 K; being at either of these bounds would in our opinion only have yielded marginally satisfactory results. If we next enhance the climate sensitivity to 7 K by decreasing *λ* to 0.85 W·m^−2^·K^−1^, keeping everything else the same, we see that the model would have warmed around 0.5 K more than observed. The climate sensitivity was determined from a very long simulation with doubled CO_2_ as it depends also on the quadratic term (*a*). If we next enhance the aerosol cooling from −0.65 W m^−2^ to around −1.5 W m^−2^ we again obtain a similar overall warming. In this case the temperature is nevertheless colder than observed in the 1960s to early 2000s, which is a consequence of the temporal evolution of the aerosol forcing that increased up until the 1970s and then changed only little afterwards as greenhouse gas forcing rose more steadily in time (Figure 7). It is therefore difficult to compensate a high climate sensitivity only with strong aerosol cooling and obtain a realistic temporal evolution (Zhao et al., 2018), and the behavior seen in the two‐layer model simulation here can be seen in several of the recent CMIP6 models with high ECS (Andrews et al., 2019; Flynn & Mauritsen, 2020; Golaz et al., 2019; Held et al., 2019). The planetary imbalance in year 2011 in both cases, 0.76 W m^−2^ for the standard setup, and 0.80 W m^−2^ with high climate sensitivity and strong aerosol cooling, are close to but slightly higher than the observed estimate of 0.71 W m^−2^± 0.10 for the period 2005–2015 (Johnson et al., 2016). This leaves essentially a stronger pattern effect (Armour, 2017) as a viable option to dampen warming while retaining a reasonable temperature evolution. A stronger pattern effect (larger *ϵ*) leads to a lower transient climate response without affecting ECS.

## 6 Closing Remarks

We have documented how we tuned the MPI‐ESM1.2 global climate model to match the instrumental record warming; an endeavor which has clearly been successful. Due to the historical order of events, the choice was to do this practically by targeting an ECS of about 3 K using cloud feedbacks, as opposed to tuning the aerosol forcing. Tuning to the instrumental record explicitly is something new at the Max Planck Institute, but in a broader perspective perhaps it is not so new. For instance, in preparing MPI‐ESM we decided to not change parameters that at the time were thought to alter cloud feedbacks (Mauritsen et al., 2012), and furthermore the inclusion of parameterizations of aerosol indirect effects was long not motivated since the historical warming was perceived as reasonable without such complicated and poorly constrained effects. Thus, the distinction between tuning and model development decisions is not always clear (Hourdin et al., 2017). It is within this gray zone where we hope to bring clarity by documenting our development choices.

**Figure 8:**
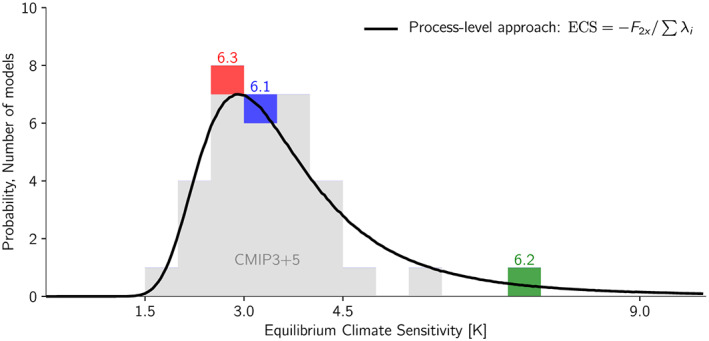
Probability distribution of ECS in CMIP3 and CMIP5 models, as tabulated in Mauritsen and Stevens (2015), shown in gray shading and for ECHAM6.1, ECHAM6.2, and ECHAM6.3 in colors. The black curve is Monte Carlo sampled forcing and feedback parameters obtained from Caldwell et al. (2016), omitting near‐identical models, and assuming the forcing, and Planck, cloud, surface albedo and water vapor plus lapse rate feedback parameters are Gaussian distributed and statistically independent.

A climate sensitivity of 7 K, as we saw it in ECHAM6.2, may seem extreme but is actually not unexpected to arise occasionally from model development. If one views climate modeling as a noisy or random process wherein development decisions lead to variations in the forcing and feedback related processes resulting in varying climate sensitivities, then the probability distribution is skewed to high values (Figure 8, Roe & Baker, 2007). Thus, in this view of climate modeling there is a small but finite chance of obtaining such high climate sensitivities. When we compare this expected‐ to the actual distribution of CMIP3 and CMIP5 models, we see a reasonable fit, but there is a lack of models with high climate sensitivities. It is only possible to speculate why this is as there can be several explanations. It may simply be that there has not been constructed enough models to expect a smooth distribution, or that the idea of Roe and Baker (2007) is not applicable to high climate sensitivities. However, it is also possible that such sensitive models have been discarded, and the anecdotal evidence given here supports this, but it is not possible to assert how widespread this practice is. In this regard it is interesting that some CMIP6 models do exhibit larger climate sensitivities than seen in CMIP3 and CMIP5; however, there is evidence that this reflects a community‐wide systematic shift in the representation of extratropical clouds, and not simply random fluctuations (Flynn & Mauritsen, 2020; Zelinka et al., 2020).

One may rightfully be concerned that we treated Earth's climate sensitivity as if it was any other observable target used during tuning, in particular given the iconic status of the 3 K best estimate first proposed by Charney et al. (1979). However, the target in the tuning was not a particular climate sensitivity, rather it was an improved match to the instrumental record, and changing the climate sensitivity was a means to that end. The acquired capability to alter the climate sensitivity within a model may, nevertheless, turn out to be useful in the quest to better constrain climate sensitivity as model versions with outlier values may be constructed and tested against instrumental or paleoclimate proxy evidence, thereby providing more confidence in methods used to infer the Earth's climate sensitivity.

## Supporting information



Supporting Information S1

## References

[jame21113-bib-0001] Andrews, T. , Andrews, M. B. , Bodas‐Salcedo, A. , Jones, G. S. , Kuhlbrodt, T. , Manners, J. , Menary, M. B. , Ridley, J. , Ringer, M. A. , Sellar, A. A. , Senior, C. A. , & Tang, Y. (2019). Forcings, feedbacks, and climate sensitivity in HadGEM3‐GC3.1 and UKESM1. Journal of Advances in Modeling Earth Systems, 11, 4377–4394. 10.1029/2019MS001866

[jame21113-bib-0002] Andrews, T. , Gregory, J. M. , Webb, M. J. , & Taylor, K. E. (2012). Forcing, feedbacks and climate sensitivity in CMIP5 coupled atmosphere‐ocean climate models. Geophysical Research Letters, 39, L09712 10.1029/2012gl051607

[jame21113-bib-0003] Armour, K. (2017). Energy budget constraints on climate sensitivity in light of inconstant climate feedbacks. Nature Climate Change, 7, 331–335. 10.1038/nclimate3278

[jame21113-bib-0004] Brient, F. , Schneider, T. , Tan, Z. , Bony, S. , Qu, X. , & Hall, A. (2016). Shallowness of tropical low clouds as a predictor of climate models response to warming. Climate Dynamics, 47, 433–449. 10.1007/s00382-015-2846-0

[jame21113-bib-0005] Caldwell, P. M. , Zelinka, M. D. , Taylor, K. E. , & Marvel, K. (2016). Quantifying the sources of inter‐model spread in equilibrium climate sensitivity. Journal of Climate, 29(2), 513–524. 10.1175/JCLI-D-15-0352.1

[jame21113-bib-0006] Cess, R. D. , Potter, G. L. , Blanchet, J. P. , Boer, G. J. , Ghan, S. J. , Kiehl, J. T. , Le Treut, H. , Li, Z.‐X. , Liang, X.‐Z. , Mitchell, J. F. B. , Morcrette, J.‐J. , Randall, D. A. , Riches, M. R. , Roeckner, E. , Schlese, U. , Slingo, A. , Taylor, K. E. , Washington, W. M. , Wetherald, R. T. , & Yagai, I. (1989). Interpretaion of cloud‐climate feedback as produced by 14 atmospheric general circulation models. Science, 245, 513–516. 17750262 10.1126/science.245.4917.513

[jame21113-bib-0007] Charney, J. G. , Arakawa, A. , Baker, J. D. , Bolin, B. , Dickinson, R. E. , Goody, R. M. , Leith, C. E. , Stommel, H. M. , & Wunsch, C. I. (1979). Carbon dioxide and climate: A scientific assessment (pp. 22). Washington, DC 10.17226/12181

[jame21113-bib-0008] Choi, Y.‐S. , Ho, C.‐H. , Park, C.‐E. , Storelvmo, T. , & Ivy, T. (2014). Influence of cloud phase composition on climate feedbacks. Journal of Geophysical Research: Atmospheres, 119, 3687–3700. 10.1002/2013JD020582

[jame21113-bib-0009] Cowtan, K. , & Way, R. G. (2014). Coverage bias in the HadCRUT4 temperature series and its impact on recent temperature trends. Quarterly Journal of the Royal Meteorological Society, 140, 1935–1944. 10.1002/qj.2297

[jame21113-bib-0010] Fiedler, S. , Stevens, B. , & Mauritsen, T. (2017). On the sensitivity of anthropogenic aerosol forcing to model‐internal variability and parameterizing a Twomey effect. Journal of Advances in Modeling Earth Systems, 9, 1942–2466. 10.1002/2017MS000932

[jame21113-bib-0011] Flynn, C. M. , & Mauritsen, T. (2020). On the climate sensitivity and historical warming evolution in recent coupled model ensembles. Atmospheric Chemistry and Physics.

[jame21113-bib-0012] Forster, P. M. , Andrews, T. , Good, P. , Gregory, J. M. , Jackson, L. S. , & Zelinka, M. (2013). Evaluating adjusted forcing and model spread for historical and future scenarios in the CMIP5 generation of climate models. Journal of Geophysical Research: Atmospheres, 118, 1139–1150. 10.1002/jgrd.50174

[jame21113-bib-0013] Geoffroy, O. , Saint‐Martin, D. , Bellon, G. , Voldoire, A. , Oliviá, D. J. , & Tytéca, S. (2013). Transient climate response in a two‐layer energy‐balance model. Part II: Representation of the efficacy of deep‐ocean heat uptake and validation for CMIP5 AOGCMs. Journal of Climate, 26, 1859–1876. 10.1175/JCLI-D-12-00196.1

[jame21113-bib-0014] Gettelman, A. , Kay, J. E. , & Shell, K. M. (2012). The evolution of climate sensitivity and climate feedbacks in the community atmospheric model. Journal of Climate, 25, 1453–1469. 10.1175/JCLI-D-11-00197.1

[jame21113-bib-0015] Giorgetta, M. A. , Jungclaus, J. , Reick, C. H. , Legutke, S. , Bader, J. , Böttinger, M. , Brovkin, V. , Crueger, T. , Esch, M. , Fieg, K. , Glushak, K. , Gayler, V. , Haak, H. , Hollweg, H.‐D. , Ilyina, T. , Kinne, S. , Kornblueh, L. , Matei, D. , Mauritsen, T. , Mikolajewicz, U. M. W. , Notz, D. , Pithan, F. , Raddatz, T. , Rast, S. , Redler, R. , Roeckner, E. , Schmidt, H. , Schnur, R. , Segschneider, J. , Six, KatharinaD. , Stockhause, M. , Timmreck, C. , Wegner, J. , Widmann, H. , Wieners, K.‐H. , Claussen, M. , Marotzke, J. , & Stevens, B. (2013). Climate and carbon cycle changes demo 1850 to 2100 in MPI‐ESM simulations for the Coupled Model Intercomparison Project phase 5. Journal of Advances in Modeling Earth Systems, 5, 572–597. 10.1002/jame.20038

[jame21113-bib-0016] Golaz, J.‐C. , Caldwell, P. M. , Van Roekel, L. P. , Petersen, M. R. , Tang, Q. , Wolfe, J. D. , Abeshu, G. , Anantharaj, V. , Asay‐Davis, X. S. , Bader, D. C. , Baldwin, S. A. , Bisht, G. , Bogenschutz, P. A. , Branstetter, M. , Brunke, M. A. , Brus, S. R. , Burrows, S. M. , Cameron‐Smith, P. J. , Donahue, A. S. , Deakin, M. E. R. C. , Evans, K. J. , Feng, Y. , Flanner, M. , Foucar, J. G. , Fyke, J. G. , Griffin, B. M. , Hannay, C. , Harrop, B. E. , Hoffman, M. J. , Hunke, E , Jacob, R. L. , Jacobsen, D. W. , Jeffery, N. , Jones, P. W. , Keen, N. D. , Klein, S. A. , Larson, V. E. , Leung, L. R. , Li, H.‐Y. , Lin, W. , Lipscomb, W. H. , Ma, P.‐L. , Mahajan, S. , Maltrud, M. E. , Mametjanov, A. , McClean, J. L. , McCoy, R. B. , Neale, R. B. , Price, S. F. , Qian, Y. , Rasch, P. J. , Reeves E. J. E. J. , Riley, W. J. , Ringler, T. D. , Roberts, A. F. , Roesler, E. L. , Salinger, A. G. , Shaheen, Z. , Shi, X. , Singh, B. , Tang, J. , Taylor, M. A. , Thornton, P. E. , Turner, A. K. , Veneziani, M. , Wan, H. , Wang, H. , Wang, S. , Williams, D. N. , Wolfram, P. J. , Worley, P. H. , Xie, S. , Yang, Y. , Yoon, J.‐H. , Zelinka, M. D. , Zender, C. S. , Zeng, X. , Zhang, C. , Zhang, K. , Zhang, Y. , Zheng, X. , Zhou, T. , & Zhu, Q. (2019). The DOE E3SM coupled model version 1: Overview and evaluation at standard resolution. Journal of Advances in Modeling Earth Systems, 11, 2089–2129. 10.1029/2018MS001603

[jame21113-bib-0017] Golaz, J.‐C. , Horowitz, L. W. , & Levy, H. (2013). Cloud tuning in a coupled climate model: Impact on 20th century warming. Geophysical Research Letters, 40, 2246–2251. 10.1002/grl.50232

[jame21113-bib-0018] Gregory, J. M. , Andrews, T. , Good, P. , Mauritsen, T. , & Forster, P. M. (2016). Small global‐mean cooling due to volcanic radiative forcing. Climate Dynamics, 47, 3979–3991. 10.1007/s00382-016-3055-1

[jame21113-bib-0019] Gregory, J. M. , & Forster, P. M. (2008). Transient climate response estimated from radiative forcing and observed temperature change. Journal of Geophysical Research, 113, D23105 10.1029/2008JD010405

[jame21113-bib-0020] Gregory, J. M. , Ingram, W. J. , Palmer, M. A. , Jones, G. S. , Stott, P. A. , Thorpe, R. B. , Lowe, J. A. , Johns, T. C. , & Williams, K. D. (2004). A new method for diagnosing radiative forcing and climate sensitivity. Geophysical Research Letters, 31, L03205 10.1029/2003GL018747

[jame21113-bib-0021] Held, I. M. , Guo, H. , Adcroft, A. , Dunne, J. P. , Horowitz, L. W. , Krasting, J. , Shevliakova, E. , Winton, M. , Zhao, M. , Bushuk, M. , Wittenberg, A. T. , Wyman, B. , Xiang, B. , Zhang, R. , Anderson, W. , Balaji, V. , Donner, L. , Dunne, K. , Durachta, J. , Gauthier, P. P. G. , Ginoux, P. , Golaz, J.‐C. , Griffies, S. M. , Hallberg, R. , Harris, L. , Harrison, M. , Hurlin, W. , John, J. , Lin, P. , Lin, S.‐J. , Malyshev, S. , Menzel, R. , Milly, P. C. D. , Ming, Y. , Naik, V. , Paynter, D. , Paulot, F. , Rammaswamy, V. , Reichl, B. , Robinson, T. , Rosati, A. , Seman, C. , Silvers, L. G. , Underwood, S. , & Zadeh, N. (2019). Structure and performance of GFDL's CM4.0 climate model. Journal of Advances in Modeling Earth Systems, 11, 3691–3727. 10.1029/2019MS001829

[jame21113-bib-0022] Held, I. M. , Winton, M. , Takahashi, K. , Delworth, T. , Zeng, F. , & Vallis, G. K. (2010). Probing the fast and slow components of global warming by returning abruptly to preindustrial forcing. Journal of Climate, 23, 2418–2427.

[jame21113-bib-0023] Hourdin, F. , Mauritsen, T. , Gettelman, A. , Golaz, J.‐C. , Balaji, V. , Duan, Q. , Folini, D. , Ji, D. , Klocke, D. , Qian, Y. , Rauser, F. , Rio, C. , Tomassini, L. , Watanabe, M. , & Williamson, D. (2017). The art and science of climate model tuning. Bulletin of the American Meteorological Society, 98, 589–602. 10.1175/BAMS-D-15-00135.1

[jame21113-bib-0024] Jiménez‐de‐la‐Cuesta, D. , & Mauritsen, T. (2019). Emergent constraints on Earth's transient and equilibrium response to doubled CO_2_ from post‐1970s global warming. Nature Geoscience, 12, 902–905. 10.1038/s41561-019-0463-y

[jame21113-bib-0025] Johnson, G. , Lyman, J. , & Loeb, N. (2016). Improving estimates of Earth's energy imbalance. Nature Climate Change, 6, 639–640. 10.1038/nclimate3043

[jame21113-bib-0026] Kiehl, J. T. (2007). Twentieth century climate model response and climate sensitivity. Geophysical Research Letters, 34, L22710 10.1029/2007GL031383

[jame21113-bib-0028] Maher, N. , Milinski, S. , Suarez‐Gutierrez, L. , Botzet, M. , Dobrynin, M. , Kornblueh, L. , Kröger, J. , Takano, Y. , Ghosh, R. , Hedemann, C. , Li, C. , Li, H. , Manzini, E. , Notz, D. , Putrasahan, D. , Boysen, L. , Claussen, M. , Ilyina, T. , Olonscheck, D. , Raddatz, T. S. B. , & Marotzke, J. (2019). The Max Planck Institute Grand Ensemble: Enabling the exploration of climate system variability. Journal of Advances in Modeling Earth Systems, 11, 2050–2069. 10.1029/2019MS001639

[jame21113-bib-0029] Mauritsen, T. , Bader, J. , Becker, T. , Behrens, J. , Bittner, M. , Brokopf, R. , Brovkin, V. , Claussen, M. , Crueger, T. , Esch, M. , Fast, I. , Fiedler, S. , Fläschner, D. , Gayler, V. , Giorgetta, M. , Goll, D. S. , Haak, H. , Hagemann, S. , Hedemann, C. , Hohenegger, C. I. T. , Jahns, T. , Jimenéz‐de‐la‐Cuesta, D. , Jungclaus, J. , Kleinen, T. , Kloster, S. , Kracher, D. , Kinne, S. , Kleberg, D. , Lasslop, G. , Kornblueh, L. , Marotzke, J. , Matei, D. , Meraner, K. , Mikolajewicz, U. , Modali, K. , Möbis, B. , Müller, W. A. , Nabel, J. E. M. S. , Nam, C. C. W. , Notz, D. , Nyawira, S.‐S. , Paulsen, H. , Peters, Ka. , Pincus, R. , Pohlmann, H. , Pongratz, J. , Popp, M. , Raddatz, T. J. , Rast, S. , Redler, R. , Reick, C. H. , Rohrschneider, T. , Schemann, V. , Schmidt, H. , Schnur, R. , Schulzweida, U. , Six, Ka. D. , Stein, L. , Stemmler, I. , Stevens, B. , von Storch, J.‐S. , Tian, F. , Voigt, A. , Vrese, P. , Wieners, K.‐H. , Wilkenskjeld, S. , Winkler, A. , & Roeckner, E. (2019). Developments in the MPI‐M Earth System Model version 1.2 (MPI‐ESM1.2) and its response to increasing CO2. Journal of Advances in Modeling Earth Systems, 11, 998–1038. 10.1029/2018MS001400 PMC7386935 32742553

[jame21113-bib-0030] Mauritsen, T. , & Stevens, B. (2015). Missing iris effect as a possible cause of muted hydrological change and high climate sensitivity in models. Nature Geoscience, 8, 346–351. 10.1038/ngeo2414

[jame21113-bib-0031] Mauritsen, T. , Stevens, B. , Roeckner, E. , Crueger, T. , Esch, M. , Giorgetta, M. , Haak, H. , Jungclaus, J. , Klocke, D. , Matei, D. , Mikolajewicz, U. , Notz, D. , Pincus, R. , Schmidt, H. , & Tomassini, L. (2012). Tuning the climate of a global model. Journal of Advances in Modeling Earth Systems, 4, M00A01 10.1029/2012MS000154

[jame21113-bib-0032] Nordeng, T. E. (1994). Extended versions of the convection parametrization scheme at ECMWF and their impact on the mean and transient activity of the model in the tropics (*206*): ECMWF.

[jame21113-bib-0033] Nuijens, L. , Medeiros, B. , Sandu, I. , & Ahlgrimm, M. (2015). Observed and modeled patterns of covariability between low‐level cloudiness and the structure of the trade‐wind layer. Journal of Advances in Modeling Earth Systems, 7, 1741–1764. 10.1002/2015MS000483

[jame21113-bib-0035] Roe, G. H. , & Baker, M. B. (2007). Why is climate sensitivity so unpredictable? Science, 318, 629–631. 10.1126/science.1144735 17962560

[jame21113-bib-0036] Schmidt, G. A. , Bader, D. , Donner, L. J. , Elsaesser, G. S. , Golaz, J.‐C. , Hannay, C. , Molod, A. , Neale, R. , & Saha, S. (2017). Practice and philosophy of climate model tuning across six U.S. modeling centers. Geoscientific Model Development, 10, 3207–3223. 10.5194/gmd-10-3207-2017 30595813 PMC6309528

[jame21113-bib-0037] Sherwood, S. C. , Bony, S. , & Dufresne, J.‐L. (2014). Spread in model climate sensitivity traced to atmospheric convective mixing. Nature, 505, 37–42. 10.1038/nature12829 24380952

[jame21113-bib-0038] Stevens, B. , Fiedler, S. , Kinne, S. , Peters, K. , Rast, S. , Müsse, J. , Smith, S. J. , & Mauritsen, T. (2017). MACv2‐SP: A parameterization of anthropogenic aerosol optical properties and an associated Twomey effect for use in CMIP6. Geoscientific Model Development, 10(1), 433–452. 10.5194/gmd-10-433-2017

[jame21113-bib-0039] Stevens, B. , Giorgetta, M. , Esch, M. , Mauritsen, T. , Crueger, T. , Rast, S. , Salzmann, M. , Schmidt, H. , Bader, J. , Block, K. , Brokopf, R. , Fast, I. , Kinne, S. , Kornblueh, L. , Lohmann, U. , Pincus, R. , Reichler, T. , & Roeckner, E. (2013). Atmospheric component of the MPI‐M Earth System Model: ECHAM6. Journal of Advances in Modeling Earth Systems, 5, 146–172. 10.1002/jame.20015

[jame21113-bib-0040] Sundqvist, H. , Berge, E. , & Kristjansson, J. E. (1989). Condensation and cloud parameterization studies with a mesoscale numerical weather prediction model. Monthly Weather Review, 117, 1641–1657.

[jame21113-bib-0041] Tiedtke, M. (1989). A comprehensive mass flux scheme for cumulus parameterization in large‐scale models. Monthly Weather Review, 117, 1779–1800.

[jame21113-bib-0042] Winton, M. , Takahashi, K. , & Held, I. M. (2010). Importance of ocean heat uptake efficacy to transient climate change. Journal of Climate, 23, 2333–2344.

[jame21113-bib-0043] Zelinka, M. D. , Myers, T. A. , McCoy, D. T. , Po‐Chedley, S. , Caldwell, P. M. , Ceppi, P. , Klein, S. A. , & Taylor, K. E. (2020). Causes of higher climate sensitivity in CMIP6 models. Geophysical Research Letters, 47, e2019GL085782 10.1029/2019GL085782

[jame21113-bib-0044] Zhao, M. (2014). An investigation of the connections among convection, clouds, and climate sensitivity in a Global Climate Model. Journal of Climate, 27, 1845–1862.

[jame21113-bib-0045] Zhao, M. , Golaz, J.‐C. , Held, I. M. , Guo, H. , Balaji, V. , Benson, R. , Chen, J.‐H. , Chen, X. , Donner, L. J. , Dunne, J. P. , Dunne, K. , Durachta, J. , Fan, S.‐M. , Freidenreich, S. M. , Garner, S. T. , Ginoux, P. , Harris, L. M. , Horowitz, L. W. , Krasting, J. P. , & Langenhorst, A. R. (2018). The GFDL global atmosphere and land model AM4.0/LM4.0: 2. Model description, sensitivity studies, and tuning strategies. Journal of Advances in Modeling Earth Systems, 10, 735–769. 10.1002/2017MS001209

