# Supplementary material for: Tuning the MPI‐ESM1.2 Global Climate Model to Improve the Match With Instrumental Record Warming by Lowering Its Climate Sensitivity
Source: J Adv Model Earth Syst. 2020 May 1;12(5):e2019MS002037. doi: 10.1029/2019MS002037 (PMC7375142; doi:10.1029/2019MS002037)
Supplement: Supplementary file 1 — Supporting Information S1 [file JAME-12-e2019MS002037-s001.pdf]

AGU PUBLICATIONS

Journal of Advances in Modeling Earth Systems

Supporting Information for

Tuning the MPI-ESM1.2 global climate model to improve the match with instrumental record warming by lowering its climate sensitivity

Thorsten Mauritsen[1] and Erich Roeckner[2]

[1]Department of Meteorology, Stockholm University, Stockholm, Sweden
[2]Max Planck Institute for Meteorology, Hamburg, Germany

Contents of this file

Tables S1 to S4

Introduction

This supplement contains original notes taken during the tuning of MPI-ESM1.2, the equivalent to laboratory notes, and they are intentionally left unaltered. Therefore, reading them inevitably requires some expertise. Based on these notes it is possible to gain an insight into the pathway taken during tuning and to repeat the experiments discussed in the main paper.

Table S1 provides an overview of the model parameters, and Table S2 an overview of their effect on the mean climate. Table S3 provides comparisons between experiments wherein a single parameter is changed. Finally, Table S4 contains results from the *amip4K* experiments and associated estimates of climate sensitivity. Based on this last table it is possible to trace and reproduce the tuning regarding climate sensitivity in MPI-ESM1.2. Figure 2 of the main paper is based on information extracted from Table S4.

Table S1. Overview of tuning parameters.

Parameter	Explanation	Comments
cmfctop	Fractional detrainment of convective cloud water above the zero-buoyancy layer	Increased cmfctop ➔ less clouds and more radiative input (sw+lw)
cprcon	Determines the conversion of convective cloud water to convective precipitation	Decreased cprcon ➔ more cloud water, less sw absorption, less outgoing lw radiation (olr), but the sw cooling dominates; weaker water cycle
zinhoml	Factor ≤ 1 applied to the cloud liquid water content to represent an inhomogeneous distribution of cloud water within the grid box	Decreased zinhoml ➔ more sw absorption but also enhanced olr (the sw effect dominates)
zinhomi	as above, but for ice clouds	Same as for zinhoml, but here the lw effect is dominating
zn1	Cloud droplet number concentration [cm^{-3}] in the upper troposphere	Hardly any impact on the results when varied in the range of 20 to 50.
zn2	Cloud droplet number concentration [cm^{-3}] in the boundary layer	Reducing zn2 has two effects: increased sw absorption by lowering the cloud optical depth and, second, enhanced autoconversion resulting in less cloud water and enhanced sw absorption. Lowering zn2 over sea tends to weaken the water cycle over land. The reason is unclear. Over land, increasing (decreasing) zn2 is weakening (enhancing) the water cycle.
nex	Determines the vertical profile of the relative humidity threshold for cloud formation between the near-surface value (crs=0.9) and that in the upper troposphere (crt=0.7). For nex=4, crt is already reached around 600 hPa. For nex=1, crt around 200 hPa.	Smaller nex ➔ less cloud cover above the boundary layer. The impact of lowering nex is almost additive: 4 ➔ 1 ≈ 4 ➔ 2 + 2 ➔ 1 ➔ More sw absorption, less cloud water and enhanced water cycle over land because more water vapor is available for the transport to land (by the trade winds, for example)
crt	Relative humidity threshold for cloud formation in the upper troposphere	Increasing crt ➔ decreasing cloud cover (especially high clouds)
crs	Relative humidity threshold for cloud formation in the lowest model level	Increasing crs ➔ decreasing cloud cover (especially low clouds)
csecfrl	Threshold determing the separation between cloud liquid water and cloud ice	Increasing csecfrl ➔ increasing cloud liquid water
cvtfall	Determines the fall speed of cloud ice	Decreased cvtfall ➔ more ice clouds and less OLR
csatsc	Relative humidity at which cloud fraction = 1 in a layer below a low-inversion (< 2000m).	csatsc < 1 ➔ more low-level stratus ➔ less sw absorption, enhanced water cycle. Note that enhanced cloud formation above the boundary layer (e.g., by increasing nex) leads to a weaker water cycle

Table S2. Overview of tuning parameters and their influence on climate sensitivity.

Impact of parameter changes on total feedback and (estimated) climate sensitivity to CO2-doubling
Red: Parameter choices in the current coupled model run (mbe0606)

parameter	change	Δ feedback factor [$Wm^{-2}K^{-1}$]	Δ sensitivity [K]
entrscv	3.e-4 → 3.e-3	-0.91 [-0.85 → -1.76]	-3.5
entrscv<br>+ cminbuoy	3.e-4 → 1.e-3<br>0.1 → 0.2	-0.82 [-0.85 → -1.67]	-3.1
csatsc	1.0 → 0.7	-0.18 [-1.56 → -1.74]	-0.7
csatsc	1.0 → cloud fraction = 1 in layer below inversion	-0.23 [-1.56 → -1.79]	-0.9
csecfrl	5.e-6 → 5.e-7	-0.15 [-1.58 → -1.73]	-0.6
csecfrl	1.e-5 → 5.e-6	-0.05 [-1.51 → -1.56]	-0.2
nex	1 → 2	-0.05 [-1.60 → -1.65]	-0.2
nex<br>+ crt	1 → 2<br>0.80 → 0.75	-0.12 [-1.44 → -1.56]	
zinhoml1<br>+ zinhoml2	0.7 → 0.8<br>0.7 → 0.4	-0.05 [-1.18 → -1.23]	-0.2
cprcon	2.e-4 → 3.e-4<br>coupled mbe0606: 2.5e-4	-0.03 [-1.74 → -1.77]	-0.1
crs		~ 0	

Parameter	Explanation
entrscv	entrainment rate for shallow convection
cminbuoy	minimum standard dev. of near-surface virtual potential temperature used for triggering convection
csatsc	relative humidity at which cloud fraction = 1 in a layer below a low-level inversion (< 2000m).
csecfrl	threshold determining the separation between cloud liquid water and cloud ice: larger value gives more liquid water
crt	relative humidity threshold for cloud formation in the upper troposphere
nex	determines the vertical profile of the relative humidity threshold for cloud formation between the near-surface value (crs) and that in the upper troposphere (crt) with larger nex giving a steeper profile
zinhoml1	factor ≤ 1 applied to the cloud liquid water content to represent an inhomogeneous distribution of cloud water within the grid box (for all cloud types except those generated by shallow convection)
zinhoml2	same as zinhoml1 but for shallow convection and LWP above cloud top < 20% of total LWP (single cloud layer)
cprcon	determines the conversion of convective cloud water to convective precipitation
crs	Relative humidity threshold for cloud formation in the lowest model level

Table S3. Single parameter changes between experiments and their influence on mean climate.

Impact of parameter changes on global variables (top-of-atmosphere radiation, cloud, water cycle)

parameter	change	toa sw rad (W/m^2)	toa lw rad (W/m^2)	toa net rad (W/m^2)	cloud cover (%)	liquid water (%)	precip (%)	precip (land) (%)	runoff (%)
cmfctop	0.21 → 0.3	0.3	0.2	0.5	-0.2	-2.8	-0.2	2.3	0.2
cprcon	2E-4 → 1.0E-4	-4.6	4.0	-0.6	2.1	8.3	-2.6	-6.7	-6.7
cprcon	2E-4 → 1.5E-4	-2.1	1.4	-0.7	1.1	4.5	-1.0	-1.9	-1.7
cprcon	2E-4 → 2.5E-4	0.7	-0.6	0.1	-0.3	-1.9	0.2	-1.1	-4.9
zinhoml, i	.77/.8 → .6/.7	5.4	-1.5	3.9	-0.8	-1.1	0.8	2.0	0.6
zn2 (land)	220 → 180	0.1	-0.1	0	-0.1	-0.2	0.4	2.6	7.8
zn2 (land)	180 → 300	-0.9	0.6	-0.3	0.7	5.4	-0.2	-6.2	-11.3
zn2 (sea)	80 → 60	3.1	-0.1	3.0	-0.5	-12.4	-0.3	-3.6	-11.3
zn2 (sea)	80 → 70	0.6	0.1	0.7	-0.3	-2.0	-0.2	-1.9	0.4
zn2 (sea)	70 → 60	1.2	0	1.2	-0.1	-5.6	-0.2	-2.0	-1.4
zn2 (sea)	60 → 50	1.6	0.1	1.7	-0.1	-4.7	-0.5	0.5	4.4
zn2 (sea)	70 → 65	0.6	0.1	0.7	-0.2	-2.3	0.0	-0.2	-0.2
crt	0.7 → 0.85	9.5	-2.2	7.3	-2.7	-23.8	0.2	18.2	24.7
crt	0.7 → 0.80	2.2	-0.8	1.4	-0.5	-5.8	-0.4	5.1	13.1
crt	0.75 → 0.85	1.7	-0.5	1.2	-0.3	-4.2	-0.1	6.0	9.8
crt	0.7 → 0.75	3.0	-0.3	2.7	-0.9	-11.6	0.6	2.5	5.4
nex	4 → 1	5.3	-1.0	4.3	-1.9	-25.3	0.4	14.2	29.7
nex	4 → 2	3.2	-0.2	3.0	0.1	-11.3	1.2	8.5	13.0
nex	2 → 1	3.1	-0.6	2.5	-1.3	-13.0	-0.6	4.2	7.5
nex	4 → 3	1.7	0	1.7	-0.8	-7.4	-0.1	3.9	7.5
nex	3 → 2	1.3	-0.2	1.1	-0.6	-6.5	0.2	3.7	2.4
nex<br>+ crt	2 → 4<br>75 → 70	-6.0	0.6	5.4	2.3	30.1	0.5	-9.4	-13.8
cminbuoy	0.1 → 0.5	3.9	-0.5	3.4	-3.5	-3.6	-1.2	-2.0	-4.4
cvtfall	3.29 → 3.0	-0.3	0.8	0.5	0.4	0.2	-0.2	4.5	7.6
cvtfall	3.29 → 2.5	-0.7	1.9	1.2	1.2	-3.0	-0.6	2.9	1.5
csatsc	1.0 ... 0.7	-1.9	0.1	-1.8	1.8	3.2	1.1	1.7	3.9
entrscv	3.E-4 → 3.E-3	-2.3	-0.0	-2.3	-0.9	+12.0	-0.3	-4.0	-4.6

Table S3 continued. Single parameter changes between experiments and their influence on mean climate.

Impact of parameter changes on global variables (top-of-atmosphere radiation, cloud, water cycle)

parameter	change	toa sw rad (W/m^2)	toa lw rad (W/m^2)	toa net rad (W/m^2)	cloud cover (%)	liquid water (%)	precip (%)	precip (land) (%)	runoff (%)
cmfctop	0.21 → 0.3	0.3	0.2	0.5	-0.2	-2.8	-0.2	2.3	0.2
cprcon	2E-4 → 1.0E-4	-4.6	4.0	-0.6	2.1	8.3	-2.6	-6.7	-6.7
cprcon	2E-4 → 1.5E-4	-2.1	1.4	-0.7	1.1	4.5	-1.0	-1.9	-1.7
cprcon	2E-4 → 2.5E-4	0.7	-0.6	0.1	-0.3	-1.9	0.2	-1.1	-4.9
zinhoml, i	.77/.8 → .6/.7	5.4	-1.5	3.9	-0.8	-1.1	0.8	2.0	0.6
zn2 (land)	220 → 180	0.1	-0.1	0	-0.1	-0.2	0.4	2.6	7.8
zn2 (land)	180 → 300	-0.9	0.6	-0.3	0.7	5.4	-0.2	-6.2	-11.3
zn2 (sea)	80 → 60	3.1	-0.1	3.0	-0.5	-12.4	-0.3	-3.6	-11.3
zn2 (sea)	80 → 70	0.6	0.1	0.7	-0.3	-2.0	-0.2	-1.9	0.4
zn2 (sea)	70 → 60	1.2	0	1.2	-0.1	-5.6	-0.2	-2.0	-1.4
zn2 (sea)	60 → 50	1.6	0.1	1.7	-0.1	-4.7	-0.5	0.5	4.4
zn2 (sea)	70 → 65	0.6	0.1	0.7	-0.2	-2.3	0.0	-0.2	-0.2
crt	0.7 → 0.85	9.5	-2.2	7.3	-2.7	-23.8	0.2	18.2	24.7
crt	0.7 → 0.80	2.2	-0.8	1.4	-0.5	-5.8	-0.4	5.1	13.1
crt	0.75 → 0.85	1.7	-0.5	1.2	-0.3	-4.2	-0.1	6.0	9.8
crt	0.7 → 0.75	3.0	-0.3	2.7	-0.9	-11.6	0.6	2.5	5.4
nex	4 → 1	5.3	-1.0	4.3	-1.9	-25.3	0.4	14.2	29.7
nex	4 → 2	3.2	-0.2	3.0	0.1	-11.3	1.2	8.5	13.0
nex	2 → 1	3.1	-0.6	2.5	-1.3	-13.0	-0.6	4.2	7.5
nex	4 → 3	1.7	0	1.7	-0.8	-7.4	-0.1	3.9	7.5
nex	3 → 2	1.3	-0.2	1.1	-0.6	-6.5	0.2	3.7	2.4
nex<br>+ crt	2 → 4<br>75 → 70	-6.0	0.6	5.4	2.3	30.1	0.5	-9.4	-13.8
cminbuoy	0.1 → 0.5	3.9	-0.5	3.4	-3.5	-3.6	-1.2	-2.0	-4.4
cvtfall	3.29 → 3.0	-0.3	0.8	0.5	0.4	0.2	-0.2	4.5	7.6
cvtfall	3.29 → 2.5	-0.7	1.9	1.2	1.2	-3.0	-0.6	2.9	1.5
csatsc	1.0 ... 0.7	-1.9	0.1	-1.8	1.8	3.2	1.1	1.7	3.9
entrscv	3.E-4 → 3.E-3	-2.3	-0.0	-2.3	-0.9	+12.0	-0.3	-4.0	-4.6

Table S3 continued. Single parameter changes between experiments and their influence on mean climate.

new: since dec. 2013									
parameter	change	toa sw rad (W/m^2)	toa lw rad (W/m^2)	toa net rad (W/m^2)	cloud cover (%)	liquid water (%)	precip (%)	precip (land) (%)	runoff (%)
zinhoml1<br>zinhoml2<br>zinhomi	0.8 (0.6)<br>+ 0.4 (0.6)<br>+ 0.8 (0.7)	1.3	0.7	2.1	-0.4	-0.8	-1.0	0.5	8.2
zinhoml1<br>zinhoml2<br>(ktype=2)<br>zinhomi	0.8 (0.6)<br>+ 0.4 (0.6)<br><br>+ 0.8 (0.7)	-0.3	1.0	0.7	-0.3	-1.6	-1.2	-4.5	-0.8
zinhoml1	0.75 (0.8)	1.1	-0.2	0.9	-0.4	-1.5	-0.2	1.5	5.4
zinhoml1	0.7 (0.8)	1.4	-0.3	1.1	-0.4	-1.0	0.2	1.9	2.8
zinhoml2<br>(ktype=2)	0.8 (0.4)	-3.7	0.3	-3.4	0.5	-0.8	-0.3	-4.2	-8.0
zinhoml2	0.4;ratio=4<br>(0.4)	-1.8	0.0	-1.8	0.1	-1.6	-0.5	-3.6	-6.8
zinhoml1<br>zinhoml2:<br>ktype=2;<br>ratio=4	0.7 (0.8)<br>0.7 (0.4)	0.3	0	0.3	0.1	0.3	0	1.5	0.4
cmfctop	0.25 (0.20)	0.6	0.1	0.7	-0.3	-0.7	0.1	-0.4	-0.2
cmfctop	0.30 (0.25)	0.4	-0.1	0.3	-0.3	-1.5	-0.5	-1.5	-0.4
cminbuoy	0.30 (0.20)	0.3	-0.2	0.1	-0.6	0.5	0.1	0.7	1.0
csecfrl	5.e-6 (5.e-7)	-2.6	0.4	-2.2	0.2	12.3	0.5	1.9	3.1
crt	0.80 (0.75)	2.4	-0.4	2.0	-0.8	-7.6	-0.5	1.5	9.8
zinhoml1	0.75 (0.70)	-0.8	0.4	-0.4	0.3	0.4	-0.4	0.5	0
zinhoml1	0.80 (0.70)	-1.5	0.2	-1.3	0.2	-0.7	-0.3	-2.4	-2.1
zn2(sea)	0.70 (0.65)	-0.7	0	-0.7	0.1	2.6	0.4	-0.3	-2.9
cvtfall	2.5 (3.0)	-0.2	1.3	1.1	0.9	-3.9	-0.9	-1.2	-1.5

Table S3 continued. Single parameter changes between experiments and their influence on mean climate.

Parameter	change	toa sw rad (W/m^2)	toa lw rad (W/m^2)	toa net rad (W/m^2)	cloud cover (%)	liquid water (%)	precip (%)	precip (land) (%)	runoff (%)
10 years (T31L31)									
csecfrl	1.e-6 (5.e-6)	1.43	-0.30	1.13	-0.03	-12.90	0.14	1.95	0.55
csecfrl	2.e-6 (5.e-6)	0.93	-0.14	0.80	-0.04	-11.55	0.00	1.33	0.78
cinv	0.5 (0.25)	-0.42	0.06	-0.36	0.36	0.39	0.07	-0.47	-1.81
csatsc	0.5 (0.7)	-0.44	0.08	-0.37	0.43	1.89	0.45	0.58	0.00
csatsc	0.1 (0.7)	-1.12	0.21	-0.91	1.08	3.96	0.35	1.37	1.31
zinhoml3	0.4 (0.6)	1.40	-0.06	1.35	-0.02	1.03	0.60	4.64	5.73
crt<br>+ nex	0.85 (0.80)<br>1 (2)	2.66	-0.54	2.12	-1.16	-5.52	0.10	3.39	1.66
cvtfall	2.5 (3.0)	-0.40	0.99	0.60	1.16	-1.15	-0.63	1.11	1.91
cvtfall<br>+ zn2(sea)	2.0 (2.5)<br>80 (65)	-2.59	1.55	-1.04	1.66	7.86	-0.95	2.14	3.48
cmfctop	0.20 (0.25)	-1.22	0.16	-1.06	0.61	3.04	0.21	-0.81	-2.07
crt<br>+ nex<br>+ cvtfall<br>+ cmfctop<br>+ zn2(sea)	0.85 (0.80)<br>1 (2)<br>2.0 (3.0)<br>0.20 (0.25)<br>80 (65)	-1.55	2.17	0.62	2.26	3.79	-1.26	5.92	4.99
crs	0.95 (0.90)	5.40	-0.69	4.71	-3.28	-5.36	-1.41	1.34	2.88
crs	0.92 (0.91)	0.87	-0.19	0.68	-0.60	-2.17	-0.03	1.24	2.59
crs	0.92 (0.913)	0.66	-0.08	0.58	-0.42	-1.81	-0.17	0.21	-1.32
zorvari<br>+ zdrexp	200 (100)<br>5.0 (1.5)	-0.13	-0.03	-0.16	0.11	0.62	0.25	0.05	-3.93
T63L47 30 years									
entrscv	3.e-3 (3.e-4)	-2.30	-0.03	-2.33	-0.91	11.90	-0.30	-4.04	-4.59
zn2(sea)	65 (70)	0.42	0.03	0.45	0.04	-2.06	0.03	-0.05	-0.57

Table S4. Complete experiment documentation of climate sensitivity estimates displayed in manuscript Figure 2.

Feedback parameter estimated from SST+4K AMIP experiments (model parameter on next page)

Radiative flux	echam6.1	echam6.2	entrscv	mbe0507	mbe0519	mbe0542*	mbe0544	mbe0546	mbe0548	mbe0550
Total	-1.64	-0.85	-1.76	-1.67	-1.42	-1.18	-1.23	-1.24	-1.39	-1.21
Shortwave	0.35	0.87	-0.03	0.07	0.33	0.56	0.50	0.50	0.37	0.51
Longwave	-1.98	-1.73	-1.73	-1.74	-1.75	-1.74	-1.73	-1.74	-1.76	-1.72
Clear-sky SW	0.32	0.31	0.32	0.32	0.33	0.35	0.33	0.34	0.33	0.35
Clear-sky LW	-2.06	-2.03	-2.03	-2.02	-2.02	-1.96	-1.97	-1.97	-1.99	-1.97
CRE SW	0.02	0.57	-0.35	-0.25	0.00	0.21	0.16	0.16	0.03	0.16
CRE LW	0.08	0.30	0.30	0.29	0.26	0.22	0.24	0.24	0.23	0.25
CRE Total	0.10	0.87	-0.05	0.04	0.26	0.43	0.40	0.40	0.26	0.41

* echam version of pictl000m

Radiative flux	mbe0542*	mbe0552	mbe0554	mbe0556	mbe0558	mbe0560	mbe0562
Total	-1.18	-1.33	-1.22	-1.33	-1.18	-1.29	-1.46
Shortwave	0.56	0.34	0.52	0.41	0.59	0.42	0.26
Longwave	-1.74	-1.67	-1.74	-1.74	-1.75	-1.71	-1.73
Clear-sky SW	0.35	0.33	0.34	0.35	0.34	0.34	0.35
Clear-sky LW	-1.96	-1.96	-1.97	-2.01	-1.98	-1.98	-2.04
CRE SW	0.21	0.02	0.18	0.06	0.25	0.08	-0.08
CRE LW	0.22	0.29	0.23	0.27	0.20	0.27	0.31
CRE Total	0.43	0.31	0.41	0.33	0.45	0.35	0.23

Table S4 continued. Complete experiment documentation of climate sensitivity estimates displayed in manuscript Figure 2.

Comparison of parameters in ECHAM6.1 and ECHAM6.2 (T63L47)

Module/Routine	Parameter	6.1	6.2	entrscv	mbe507	mbe519	mbe542 pictl000m	mbe544	mbe546	mbe548	mbe550	mbe552	mbe554
mo_time_control	timestep	600					450						
mo_cumulus_flux	cmfctop	0.21	0.20			0.25	0.30		0.20	0.30			
“	cprcon	2e-4					2e-4						
“	entrpen	1e-4					1e-4				2e-4	1e-4	
“	entrscv	3e-4		3e-3	1e-3		1e-3			5e-3	1e-3		
“	cbfac	1.0			2.0		1.0						2.0
“	cminbuoy	0.1			0.2		0.2						
“	cmaxbuoy	1.0					1.0						
mo_echam_cloud_ params	crs	0.9					0.905				0.9	0.9	0.9
	crt	0.7	0.75				0.8						
“	nex	4	2				1						
“	cvtfall	3.29	3.0				2.5						
“	csecfrl	5e-7				5e-6	1e-5					5.e-7	1.e-5
mo_cloud_optics	zinhoml1	0.77	0.6			0.8	0.7	0.8	0.7				
“	zinhoml2	0.77	0.6			0.4*	0.7	0.4**	0.7				
“	zinhomi	0.8	0.7			0.8	0.8						
physc	zn1	50	20										
“	zn2(sea)	80	70			65	80		70	80			
“	zn2(land)	220	180										
mo_ssodrag	gstd	100	1.0				1.0						
	gpicmea	400	1.0				1.0						
	gkwake	0.5	1.0				1.0						
	gkdrag	0.5	0.2				0.2						

Changes w.r.t. mbe542

Changes w.r.t. mbe507

* ktype = 2 (shallow convection)

** ktype = 2 and LWP(above cloud top) < 20% of total LWP (clwprat = 4.0)

integer land/sea mask used in mbe0542 etc.

Table S4 continued. Complete experiment documentation of climate sensitivity estimates displayed in manuscript Figure 2.

Module/Routine	Parameter	6.1	6.2	entrscv	mbe507	mbe519	mbe542 pictl000m	mbe556	mbe558	mbe560	mbe562
mo_time_control	timestep	600					450				
mo_cumulus_flux	cmfctop	0.21	0.20			0.25	0.30				
“	cprcon	2e-4					2e-4				
“	entrpen	1e-4					1e-4				
“	entrscv	3e-4		3e-3	1e-3		1e-3				
“	cbfac	1.0			2.0		1.0				
“	cminbuoy	0.1			0.2		0.2				
“	cmaxbuoy	1.0					1.0				
mo_echam_cloud_ params	crs	0.9					0.905	0.9	0.9	0.93	0.95
	crt	0.7	0.75				0.8	0.75	0.8	0.75	0.7
“	nex	4	2				1	2	1	2	4
“	cvtfall	3.29	3.0				2.5		3.0	2.5	
“	csecfrl	5e-7				5e-6	1e-5				
mo_cloud_optics	zinhoml1	0.77	0.6			0.8	0.7				
“	zinhoml2	0.77	0.6			0.4*	0.7				
“	zinhomi	0.8	0.7			0.8	0.8				
physc	zn1	50	20								
“	zn2(sea)	80	70			65	80				
“	zn2(land)	220	180								
mo_ssodrag	gstd	100	1.0				1.0				
	gpicmea	400	1.0				1.0				
	gkwake	0.5	1.0				1.0				
	gkdrag	0.5	0.2				0.2				

Changes w.r.t. mbe542
Changes w.r.t. mbe507

* ktype = 2 (shallow convection)
** ktype = 2 and LWP(above cloud top) < 20% of total LWP (clwprat = 4.0)

integer land/sea mask used in mbe0542 etc.

Table S4 continued. Complete experiment documentation of climate sensitivity estimates displayed in manuscript Figure 2.

Feedback parameter estimated from SST+4K AMIP experiments (model parameter on next page)

Radiative flux	echam6.1	echam6.2	entrscv	mbe542*	mbe564	mbe566*	MP	mbe570	mbe572	mbe574	mbe576
Total	-1.64	-0.85	-1.76	-1.18	-1.74	-1.73	-1.48	-1.58	-1.65	-1.60	-1.46
Shortwave	0.35	0.87	-0.03	0.56	-0.03	-0.05	0.42	0.10	0.05	0.11	0.24
Longwave	-1.98	-1.73	-1.73	-1.74	-1.70	-1.68	-1.90	-1.68	-1.70	-1.71	-1.70
Clear-sky SW	0.32	0.31	0.32	0.35	0.33	0.33	0.34	0.34	0.32	0.32	0.33
Clear-sky LW	-2.06	-2.03	-2.03	-1.96	-2.00	-1.99	-2.04	-1.99	-1.99	-1.99	-1.97
CRE SW	0.02	0.57	-0.35	0.21	-0.36	-0.38	0.08	-0.24	-0.27	-0.21	-0.09
CRE LW	0.08	0.30	0.30	0.22	0.30	0.31	0.14	0.31	0.29	0.28	0.27
CRE Total	0.10	0.87	-0.05	0.43	-0.06	-0.07	0.21	0.07	0.02	0.07	0.17

* echam version of pictl000m
* echam version of pictl000o

Table S4 continued. Complete experiment documentation of climate sensitivity estimates displayed in manuscript Figure 2.

Comparison of parameters in ECHAM6.1 and ECHAM6.2 (T63L47)

Module/Routine	Parameter	6.1	6.2	entrscv	mbe542 pictl000m	mbe564	mbe566	MP	mbe570	mbe572	mbe574	mbe576
mo_time_control	timestep	600			450	450						
mo_cumulus_flux	cmfctop	0.21	0.20		0.30	0.20						
“	cprcon	2e-4			2e-4	2e-4						
“	entrpen	1e-4			1e-4	1e-4						
“	entrscv	3e-4		3e-3	1e-3	3e-3						
“	cbfac	1.0			1.0	1.0						
“	cminbuoy	0.1			0.2	0.2						
“	cmaxbuoy	1.0			1.0	1.0						
convective detrainment	liquid + ice							liquid only				
mo_echam_cloud_ params	crs	0.9			0.905	0.90	0.96			0.94	0.915	0.93
	crt	0.7	0.75		0.8	0.75				0.8	0.8	0.8
“	nex	4	2		1	2					1	1
“	cvtfall	3.29	3.0		2.5	2.5						
“	csecfrl	5e-7			1e-5	5.e-7			5e-6	5e-7		5e-6
mo_cloud_optics	zinhoml1	0.77	0.6		0.7	0.8						
“	zinhoml2	0.77	0.6		0.7	0.4*						
“	zinhomi	0.8	0.7		0.8	0.8						
physc	zn1	50	20		20	20						
“	zn2(sea)	80	70		80	80						
“	zn2(land)	220	180		180	180						
mo_ssodrag	gstd	100	1.0		1.0	1.0						
	gpicmea	400	1.0		1.0	1.0						
	gkwake	0.5	1.0		1.0	1.0						
	gkdrag	0.5	0.2		0.2	0.2						

Changes w.r.t. mbe566 = new reference run with low sensitivity as used in pictl000o (except for crs = 0.947)

* ktype = 2 and LWP(above cloud top) < 20% of total LWP (clwprat = 4.0)

integer land/sea mask used in mbe0542 etc.

Table S4 continued. Complete experiment documentation of climate sensitivity estimates displayed in manuscript Figure 2.

Feedback parameter estimated from SST+4K AMIP experiments (model parameter on next page)

Radiative flux	6.1	mbe542*	mbe564	mbe566*	MP	ER	mbe570	mbe572	mbe574	mbe576	mbe598
Total	-1.64	-1.18	-1.74	-1.73	-1.48	-1.50	-1.58	-1.65	-1.60	-1.46	-1.56
Shortwave	0.35	0.56	-0.03	-0.05	0.42	0.37	0.10	0.05	0.11	0.24	0.13
Longwave	-1.98	-1.74	-1.70	-1.68	-1.90	-1.87	-1.68	-1.70	-1.71	-1.70	-1.69
Clear-sky SW	0.32	0.35	0.33	0.33	0.34	0.34	0.34	0.32	0.32	0.33	0.34
Clear-sky LW	-2.06	-1.96	-2.00	-1.99	-2.04	-2.02	-1.99	-1.99	-1.99	-1.97	-2.00
CRE SW	0.02	0.21	-0.36	-0.38	0.08	0.03	-0.24	-0.27	-0.21	-0.09	-0.21
CRE LW	0.08	0.22	0.30	0.31	0.14	0.15	0.31	0.29	0.28	0.27	0.30
CRE Total	0.10	0.43	-0.06	-0.07	0.21	0.18	0.07	0.02	0.07	0.17	0.09

* echam version of pictl000m
* echam version of pictl000o

MP: only liquid water detrained
ER: cloud ice detrained at temperatures T < cthomi = 238K, otherwise liquid water

Table S4 continued. Complete experiment documentation of climate sensitivity estimates displayed in manuscript Figure 2.

Comparison of parameters in ECHAM6.1 and ECHAM6.2 (T63L47)

Module/Routine	Parameter	6.1	mbe542 pictl000m	mbe564	mbe566	mbe570	mbe572	mbe574	mbe576	mbe598
mo_time_control	timestep	600	450	450						
mo_cumulus_flux	cmfctop	0.21	0.30	0.20						
“	cprcon	2e-4	2e-4	2e-4						
“	entrpen	1e-4	1e-4	1e-4						
“	entrscv	3e-4	1e-3	3e-3						
“	cbfac	1.0	1.0	1.0						
“	cminbuoy	0.1	0.2	0.2						
“	cmaxbuoy	1.0	1.0	1.0						
mo_echam_cloud_	crs	0.9	0.905	0.90	0.96		0.94	0.915	0.93	0.97
params	crt	0.7	0.8	0.75			0.8	0.8	0.8	0.75
“	nex	4	1	2				1	1	2
“	cvtfall	3.29	2.5	2.5						
“	csecfrl	5e-7	1e-5	5.e-7		5e-6	5e-7		5e-6	5e-6
mo_cloud_optics	zinhoml1	0.77	0.7	0.8						
“	zinhoml2	0.77	0.7	0.4*						
“	zinhomi	0.8	0.8	0.8						
physc	zn1	50	20	20						
“	zn2(sea)	80	80	80						
“	zn2(land)	220	180	180						
mo_ssodrag	gstd	100	1.0	1.0						
	gpicmea	400	1.0	1.0						
	gkwake	0.5	1.0	1.0						
	gkdrag	0.5	0.2	0.2						

Changes w.r.t. mbe566 = new reference run with low sensitivity as used in pictl000o (except for crs = 0.947)

* ktype = 2 and LWP(above cloud top) < 20% of total LWP (clwprat = 4.0)

integer land/sea mask used in mbe0542 etc.

Table S4 continued. Complete experiment documentation of climate sensitivity estimates displayed in manuscript Figure 2.

Feedback parameter estimated from SST+4K AMIP experiments (model parameter on next page)

Radiative flux	6.1	542	566	570	576	598	598(2)	598(3)	600(1)	600(2)	603(2)	609(2)	614
Total	-1.64	-1.18	-1.73	-1.58	-1.46	-1.51	-1.56	-1.44	-1.57	-1.74	-1.74	-1.79	-1.77
Shortwave	0.35	0.56	-0.05	0.10	0.24	0.20	0.13	0.30	0.15	-0.01	-0.03	-0.10	-0.07
Longwave	-1.98	-1.74	-1.68	-1.68	-1.70	-1.70	-1.69	-1.74	-1.72	-1.72	-1.70	-1.69	-1.70
Clear-sky SW	0.32	0.35	0.33	0.34	0.33	0.34	0.34	0.34	0.34	0.34	0.34	0.33	0.33
Clear-sky LW	-2.06	-1.96	-1.99	-1.99	-1.97	-2.01	-2.00	-1.98	-1.97	-2.02	-2.00	-2.00	-2.00
CRE SW	0.02	0.21	-0.38	-0.24	-0.09	-0.15	-0.21	-0.04	-0.20	-0.35	-0.38	-0.43	-0.40
CRE LW	0.08	0.22	0.31	0.31	0.27	0.30	0.30	0.25	0.25	0.30	0.30	0.31	0.30
CRE Total	0.10	0.43	-0.07	0.07	0.17	0.15	0.09	0.20	0.05	-0.05	-0.08	-0.12	-0.10

<u>Corresponding coupled models (except for very small changes in crs)</u>:
pictl000m
pictl000o
pictl000p
pictl000q
mbe0606, mbe0624, mbe0642 (cprcon = 2.5e-4)

Table S4 continued. Complete experiment documentation of climate sensitivity estimates displayed in manuscript Figure 2.

Comparison of parameters in ECHAM6.1 and ECHAM6.2 (T63L47)

Module/Routine	Parameter	6.1	542 pictl000m	566 pictl000o	570	576	598	598(2) pictl000p	598 (3)	600(1) pictl000q	600 (2)	603(2) mbe606	609 (2)	614
mo_time_control	timestep	600	450											
mo_cumulus_flux	cmfctop	0.21	0.30	0.20										
“	cprcon	2e-4	2e-4	2e-4										3e-4
“	entrpen	1e-4	1e-4	1e-4										
“	entrscv	3e-4	1e-3	3e-3										
“	cbfac	1.0	1.0	1.0										
“	cminbuoy	0.1	0.2	0.2										
“	cmaxbuoy	1.0	1.0	1.0										
mo_echam_cloud_	crs	0.9	0.905	0.96		0.93	0.97	0.97	0.92	0.94	0.97	0.978	0.97	0.978
params	crt	0.7	0.8	0.75		0.8	0.75	0.75	0.8	0.8	0.75	0.75	0.75	0.75
“	nex	4	1	2	2	1	2	2	1	1	2	2	2	2
“	cvtfall	3.29	2.5	2.5										
“	csecfrl	5e-7	1e-5	5e-7	5e-6	5e-6	1e-5	5e-6	5e-6	5e-6	5e-6	5e-6	5e-6	5e-6
“	csatsc	1.0	1.0	1.0	1.0	1.0	1.0	1.0	1.0	0.7	0.7	0.7	****	0.7
mo_cloud_optics	zinhoml1	0.77	0.7	0.8										
“	zinhoml2	0.77	0.7	0.4*										
“	zinhomi	0.8	0.8	0.8										
physc	zn1	50	20	20										
“	zn2(sea)	80	80	80										
“	zn2(land)	220	180	180										
mo_ssodrag	gstd	100	1.0	1.0										
	gpicmea	400	1.0	1.0										
	gkwake	0.5	1.0	1.0										
	gkdrag	0.5	0.2	0.2										

* ktype = 2 and LWP(above cloud top) < 20% of total LWP (clwprat = 4.0)

integer land/sea mask used in mbe0542 etc.

**** cloud fraction is set to 1.0 in the layer below a low-level inversion
